# General Use of Chinese Herbal Products among Female Patients with Mastitis in Taiwan

**DOI:** 10.1155/2022/3876240

**Published:** 2022-03-25

**Authors:** Shu-Huey Chou, Chun-Che Huang, Ching-Heng Lin, Kun-Chang Wu, Pei-Jung Chiang

**Affiliations:** ^1^Department of Traditional Chinese Medicine, Taichung Veterans General Hospital, Taichung, Taiwan; ^2^Department of Healthcare Administration, I-Shou University, Kaohsiung, Taiwan; ^3^Department of Medical Research, Taichung Veterans General Hospital, Taichung, Taiwan; ^4^Department of Public Health, Fu-Jen Catholic University, New Taipei, Taiwan; ^5^Department of Health Care Management, National Taipei University of Nursing and Health Sciences, Taipei, Taiwan; ^6^Department of Industrial Engineering and Enterprise Information, Tunghai University, Taichung, Taiwan; ^7^School of Pharmacy, College of Pharmacy, China Medical University, Taichung, Taiwan; ^8^Graduate Institute of Chinese Medicine, School of Chinese Medicine, China Medical University, Taichung, Taiwan

## Abstract

**Objectives:**

Little information is available regarding the use of Chinese herbal medicine to treat mastitis. This study evaluated the prescription patterns of Chinese herbal medicine products in women with mastitis in Taiwan.

**Design:**

This is a population-based cross-sectional study. *Setting*. 8,531 women aged 20–49 years, who received a diagnosis of mastitis between 2004 and 2013, were identified from the Longitudinal Health Insurance Database in Taiwan. We collected data on demographic characteristics, including age, monthly insurance premium, and urbanization level. The ten most Chinese herbal medicines prescribed for mastitis were assessed, including frequency, percentage, average daily dose, and average duration of prescription. *Main outcome measures*. We analysed the ten most single Chinese herbs and Chinese herbal formulae prescribed for mastitis.

**Results:**

Overall, 437 (5.1%) women received Chinese herbal medicine to treat mastitis. Mai Men Dong (*Ophiopogon japonicus* (Thunb.) Ker Gawl.; 22.3%), Pu Gong Yin (*Taraxacum mongolicum* Hand.-Mazz.; 7.8%), and Wang Bu Liu Xing (*Vaccaria hispanica* (Mill.) Rauschert; 3.5%) were three of the most commonly prescribed single Chinese herbs for mastitis. Xian-Fang-Huo-Ming-Yin (18.2%), Jia-Wei-Xiao-Yao-San (9.1%), and Chai-Hu-Shu-Gan-San (8.4%) were three of the most commonly prescribed Chinese herbal formulae.

**Conclusion:**

Xian-Fang-Huo-Ming-Yin can clear heat, detoxify body, alleviate swelling, activate blood, and relieve pain. It was the most frequently prescribed Chinese herbal formula in patients with mastitis.

## 1. Introduction

Mastitis is an inflammation of the breast, which may or may not involve an infection. It is usually related to lactation. The World Health Organization reported that incidence of mastitis in lactating women ranged from 3% to 33% [1]; it usually occurs in the first six weeks postpartum, and the incidence gradually declines thereafter [2]. The major causes of mastitis are milk stasis and infection [1]; other factors include genetic factors, immune factors, trauma, etc. However, women with nonlactational causes of mastitis are less common [3]. A previous study reported the frequency of nonlactational mastitis among biopsies for benign breast diseases to be 3% [4].

Mastitis diagnosis is usually based on clinical symptoms and signs, such as breast pain, heat, swelling, fever, and chills [2]. In 946 breastfeeding women, Foxman et al. [5] found that the most common mastitis symptoms were breast tenderness (98%), malaise (87%), fever (82%), chills (78%), redness (78%), and a hot spot on the affected breast (62%).

Mastitis should be treated immediately, as a delay in treatment or inappropriate management can lead to breast abscess, which occurs in 5 to 11% of mastitis cases [6]. The treatments for mastitis usually include effective milk removal, counselling, as well as antibiotic and symptomatic treatment. Other therapies include the use of cabbage leaves and herbal treatment [1]. Cabbage leaves, with both antibiotic and anti-irritant properties, can reduce pain and increase breastfeeding duration [1, 7]. Gleditsiae Fructus extract has also been reported to be effective in treating mastitis [1]. Wu et al. [8] found that Taraxaci Herba, Glycyrrhizae Radix et Rhizoma, Paeoniae Radix Alba, and Citri Reticulatae Semen were the most commonly prescribed Chinese medicines for mastitis.

Mastitis and breast abscess are known as “Ru Yong” (breast carbuncle) in Chinese medicine. Ge Zhi Yu Lun, an ancient Chinese medical text, states that the breasts itself belong to the stomach meridian, and the nipples belong to the liver meridian. If a nursing mother is angry, depressed, or eats excessive amounts of greasy food, the *Qi* will stagnate and milk ducts will become blocked, causing milk stasis, which may transform into heat and possibly also an abscess. If the infant has interior heat, they might pass the heat to the mother through breastfeeding, which can cause breast lumps [9]. Chinese medicine is widely used in Taiwan. However, very few studies have investigated the application of Chinese herbal medicine (CHM) in the treatment of mastitis. Therefore, the aim of the study is to assess the patterns of CHM prescriptions to treat mastitis in women in Taiwan from the Longitudinal Health Insurance Database (LHID).

## 2. Materials and Methods

### 2.1. Data Source and Study Population

This retrospective, population-based, cross-sectional study used data from the Longitudinal Health Insurance Database (LHID), where one million beneficiaries were randomly selected from the National Health Insurance Research Database (NHIRD) in Taiwan. The LHID sample and all NHIRD enrollees had no differences in age, gender, or average insured payroll-related premiums. The LHID database contains information about outpatient visits, hospital admissions, prescriptions, disease status, and patient demographics. To protect confidentiality, all identification numbers of patients and medical institutions were encrypted and maintained by Taiwan's National Health Research Institutes before extracting and analysing data. The diagnostic codes used in the LHID were according to the International Classification of Disease, 9th Revision, Clinical Modification (ICD-9-CM) coding. In addition, this study was approved by the Institutional Review Board of Taichung Veterans General Hospital (IRB No. CE15069A-3, Taiwan), and the requirement for informed consent was waived.

**Figure 1:**
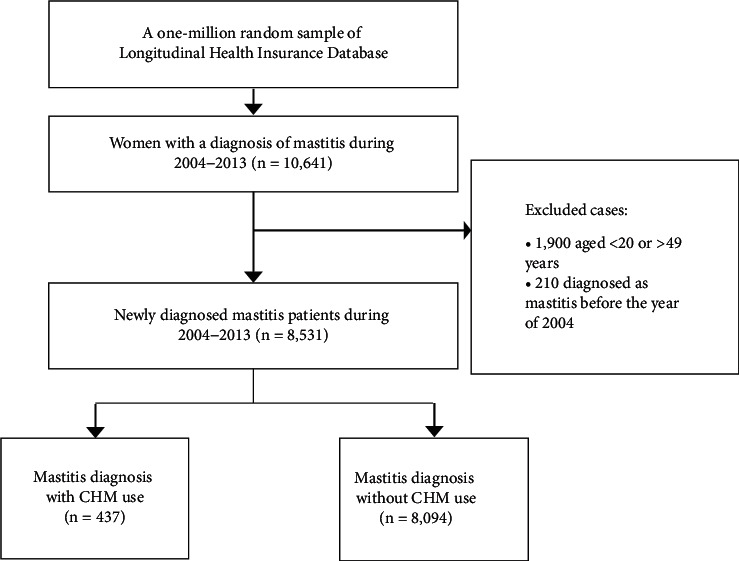
Flow diagram of sample selection. CHM, Chinese herbal medicine.

A sample of one million patients was randomly selected from the National Health Insurance Research Database (NHIRD). All patients without mastitis (ICD-9-CM code 611.0) were excluded (*n* = 989,359). A total of 10,641 women who received a diagnosis of mastitis (ICD-9-CM code 611.0) between 2004 and 2013 were identified. We excluded patients aged <20 or ≥50 years of age (*n* = 1,900) and those who were diagnosed with mastitis before 2004 (*n* = 210). The date of the initial mastitis diagnosis was defined as the index date. The final sample included 8,531 newly diagnosed patients with mastitis who were classified into those who did and did not use CHM to treat mastitis.  Figure 1 shows a flow diagram of the study selection process.

### 2.2. Chinese Herbal Medicine Use

The primary variable of interest was whether patients received CHM treatment for mastitis (ICD-9-CM code 611.0). Chinese herbal medicine products are prescribed for outpatient treatment by traditional Chinese medicine (TCM) physicians according to Taiwan's National Health Insurance program guidelines. Chinese herbal medicine use was defined as patients who had been prescribed CHM for treating mastitis at least once after the index date, whereas non-CHM use was defined as those who did not visit TCM physicians. The possible pharmacological effects of single Chinese herbs were searched from scientific literature published between July 2006 and January 2019, and the retrieval database is PubMed.

### 2.3. Variables

The demographic variables compared between the CHM and non-CHM groups were age at mastitis diagnosis, monthly insurance premium, and urbanization level.

Age was classified into groups of 20−29, 30−39, and 40−49. Individual monthly insurance premium was determined according to work salary, and premiums (in Taiwan dollars (TWD)) were classified into ≥45,801, 28,801–45,800, 15,841–28,800, <15,840, and dependent groups. The dependent group included students, stay-at-home parents, and family members without a fixed salary. The urbanization level was classified as urban, suburban, and rural.

### 2.4. Statistical Analyses

Distribution of the characteristics between patients with mastitis, with and without CHM, was examined using chi-square or Fisher's exact tests for categorical variables and Student's *t*-test for continuous variables. The prescription patterns of the 10 most prescribed single Chinese herbs and Chinese herbal formulae for mastitis treatment were analysed, including frequencies, percentages, average use duration (days/visit), and average daily dose (g). The threshold for statistical significance was set at *p* < 0.05. All analyses were conducted using SAS version 9.4 (SAS Institute Inc., Cary, NC, USA).

## 3. Results

We included 8,531 women who were newly diagnosed with mastitis between 2004 and 2013. Of these, 437 (5.1%) were CHM users and 8,094 (94.8%) were non-CHM users. The demographic characteristics of CHM users and nonusers are shown in Table 1. The mean age at diagnosis was 33.3 and 32.9 years in CHM and non-CHM users, respectively. The monthly insurance premium results revealed that over 60% of participants belong to the dependent group, which included students, stay-at-home parents, and those without a fixed salary. There was no difference in the urbanization level between the two groups.

The prescription patterns of CHM and the 10 most prescribed single Chinese herbs and Chinese herbal formulae for mastitis are presented in Tables 2 and 3, respectively. Mai Men Dong (*Ophiopogon japonicus* (Thunb.) Ker Gawl.; 22.3%), Pu Gong Yin (*Taraxacum mongolicum* Hand.-Mazz.; 7.8%), and Wang Bu Liu Xing (*Vaccaria hispanica* (Mill.) Rauschert; 3.5%) were three of the most commonly prescribed single Chinese herbs for mastitis. The most commonly prescribed Chinese herbal formula was Xian-Fang-Huo-Ming-Yin (18.2%), followed by Jia-Wei-Xiao-Yao-San (9.1%) and Chai-Hu-Su-Gan-San (8.4%). Furthermore, Jin Yin Hua (*Lonicera japonica* Thunb.), Tian Hua Fen (*Trichosanthes kirilowii* Maxim.), Zhe Bei Mu (*Fritillaria thunbergii* Miq.), Xiang Fu (*Cyperus rotundus* L.), and Pu Gong Ying (*Taraxacum mongolicum* Hand.-Mazz.) were not only part of the ten most single Chinese herbs prescribed for mastitis but also part of the ingredients of the ten most formulae prescribed for mastitis. The Chinese herbal medicine effects are summarized in Tables 2 and 3; most of these Chinese herbs can clear heat, resolve toxin, reduce swelling, and relieve pain. The possible pharmacological effects of the 10 most prescribed single Chinese herbs for mastitis are summarized in Table 2; most of these Chinese herbs have anti-inflammation and analgesic effects. The average duration for prescription of single Chinese herbs and Chinese herbal formulae are between 5.0 and 8.2 days.

## 4. Discussion

Mastitis is a common problem faced by breastfeeding women. However, this is the first study to investigate TCM prescription patterns among female patients with mastitis in Taiwan. For this, we used data from the LHID on prescriptions made by registered TCM practitioners who had been trained by the Health Promotion Administration as breastfeeding instructors. Our results showed a higher proportion of CHM use for mastitis treatment in women aged 30–39 years. This may be partially due to an increasing proportion of mothers with advanced age at childbirth and they may suffer from mastitis during breastfeeding. According to the annual report of the Health Promotion Administration, Taiwanese women gave birth to their first child at an average age of 27.4 years in 2004 and 30.5 years in 2014. In addition, since 2009, more than half of the women giving birth in Taiwan have been aged between 30 and 40 years [20].

In TCM, “Ru Yong” is caused by liver *Qi* depression, stomach heat, infections, inadequate breastfeeding, or *Qi* counterflow during pregnancy, which can lead to milk accumulation. In the initial stage of treatment, the aim is to relieve symptoms, mainly by soothing the liver and regulating *Qi*, clearing stomach fire, dispersing nodules, and letting the milk flow. If mastitis progresses to an abscess, the aim is to expel pus and toxins.

The three most prescribed Chinese herbal formulae were Xian-Fang-Huo-Ming-Yin (18.2%), Jia-Wei-Xiao-Yao-San (9.1%), and Chai-Hu-Shu-Gan-San (8.4%). The three most prescribed Chinese herbs were Mai Men Dong (22.3%), Pu Gong Yin (7.8%), and Wang Bu Liu Xing (3.5%).

Xian-Fang-Huo-Ming-Yin is a well-known Chinese herbal formula that can clear heat, detoxify body, alleviate swelling, activate blood flow, and relieve pain. It has been widely applied to treat sores, carbuncles, and abscesses. Although it is commonly prescribed for mastitis, no research has yet documented the effect of Xian-Fang-Huo-Ming-Yin on mastitis. The formula is composed of 12 single Chinese herbs. Among them, Jin Yin Hua (*Lonicera japonica* Thunb.) has been reported to have an antiviral effect [19], and Tian Hua Fen (*Trichosanthes kirilowii* Maxim.) has been reported to have anti-inflammatory effects and the ability to clear heat, alleviate swelling, and expel pus [17, 21]. Zhe Bei Mu (*Fritillaria thunbergii* Miq.) is thought to inhibit interleukin-6, interleukin-8, tumour necrosis factor-*α*, and the mitogen-activated protein kinase pathway [18]; indeed, mastitis is characterized by increased interleukin-8 concentrations in milk [22]. It is worth noting that Xian-Fang-Huo-Ming-Yin does not contain Chuan Shan Jia (*Manis pentadactyla* Linnaeus) nowadays. Though it can stimulate lactation, disperse swelling, and expel pus, excessive hunting has led to becoming endangered. In 2000, the Department of Health in Taiwan banned the use of products obtained from protected species (e.g., pangolin, bear bile, musk, and Saiga antelope horn) from medical use [23]. The committee on Chinese Medicine and Pharmacy revealed that Wang Bu Liu Xing could replace Chuan Shan Jia to help increase lactation [24]. The Dean of the American College of Traditional Chinese Medicine, Steve Given, mentioned that there are 125 alternatives for Chuan Shan Jia, depending on the diagnosis, since alternatives of TCM could be composed of various products instead of a one-to-one replacement. Moreover, what was reasonable a few decades ago may not be reasonable today [25].

Jia-Wei-Xiao-Yao-San is usually prescribed to treat insomnia, depressive disorder, anxiety disorder, and functional dyspepsia [26, 27]. It can decrease serotonin and interleukin-6 and has been reported to have an antidepressant-like effect [28, 29]. Chai-Hu-Shu-Gan-San can soothe the liver and regulate *Qi* and is also used to treat anxiety and depression, especially poststroke depression and postpartum depression, according to Yan Sun's research [13, 30]. In addition, Chai Hu (*Bupleurum chinense* DC.), Xiang Fu (*Cyperus rotundus* L.), and Chuan Xiong (*Ligusticum striatum* DC.), which are part of the composition of Chai-Hu-Shu-Gan-San, also have anti-inflammatory effects [13]. Cooklin et al. [31] investigated the link between physical health, breastfeeding problems, and maternal mood and found that the presence of breastfeeding problems was associated with poorer maternal mood. Fallon et al. [32] reviewed the relationship between postpartum anxiety and infant-feeding outcomes, and their results indicated that postpartum anxiety increases breastfeeding difficulties. Webber and Benedict [33] investigated the relationship between inflammation, breastfeeding, and postpartum depression and reported a negative correlation between postpartum depression and breastfeeding. Furthermore, stress can cause inflammation and increase the risk of depression. According to the Ge Zhi Yu Lun, the classics of traditional Chinese medicine, the liver can regulate *Qi* and is associated with emotion. Emotion may cause the stagnation of *Qi*, resulting in milk stasis and mastitis. As a result, mastitis can be treated by soothing the liver and regulating *Qi*.

When it comes to the most prescribed Chinese herbs for mastitis, many of these prescription herbs are used to rectify *Qi*, clear heat, resolve toxins, disperse swelling, and relieve pain. The possible pharmacological effects of Chinese herbs are obtained from the scientific literature by PubMed and are listed in Table 2.

Mai Men Dong nourishes Yin and generates fluid and, therefore, is used as a typical treatment for Yin deficiency, dry mouth, fluid depletion, and constipation [34]. Women's constitution transferred throughout the perinatal period. A previous study revealed that Yin-Xu constitution worsened during pregnancy and did not recover at six months postpartum [35]. The use of Mai Men Dong may also ameliorate some postpartum physical symptoms, such as sweating, thirst, and constipation [36, 37]. Furthermore, Yi Xue Qi Yuan, an ancient Chinese medical text, describes that Mai Men Dong is also used to treat lactation. In addition, Mai Men Dong had been proved to have anti-inflammation effects [10].

Pu Gong Ying can clear heat toxins and relieve swelling. It has anti-inflammatory and analgesic effects and has been used to treat upper respiratory tract infections, urinary tract infections, hepatitis, and dyspepsia [11]. Wang Bu Liu Xing is effective in activating blood circulation and reducing swelling, has analgesic and anti-inflammatory effects [12], and is used to treat female mammary gland diseases and promote lactation.

Chuan Lian Zi (*Melia azedarach* L.), Lu Lu Tong (*Liquidambar formosana* Hance), and Yu Jin (*Curcuma phaeocaulis* Valeton) have anti-inflammatory effects [14–16]. Chuan Lian Zi and Yu Jin have analgesic effects [14, 16].

In this study, we found that the mastitis rate in Taiwan was much lower compared with that reported by previous studies. This underestimation might be due to the use of health care services. In Taiwan, women with mastitis usually opt for Western medicine, breast massage, or consultation with international board-certified lactation consultants. Among these treatments, the National Health Insurance does not cover breast massage or international board-certified lactation consultants; therefore, these women were not included in the study.

This study was designed to explore the prescription of TCM for mastitis in Taiwan. We found that certain Chinese medicines have anti-inflammation and analgesic effects for treating mastitis, similar to Western medicine. Furthermore, Chinese medicine rarely causes stomach pain, diarrhoea, or indigestion and may be a possible alternative for treating mastitis.

## 5. Limitations

There are two limitations to this study. First, due to the feature of the LHID, we could not differentiate the aetiology of mastitis. Nevertheless, the 10 most prescribed CHM products were compliant with the clinical treatment of lactation mastitis. Second, mastitis is diagnosed by clinical symptoms and signs, and most patients had a good prognosis after receiving appropriate treatment, so it is hard to evaluate the efficacy of the treatment.

## 6. Future Perspectives and Priorities

This study discussed the prescription patterns of Chinese herbal medicine products in women with mastitis in Taiwan. However, a well-conducted, randomized controlled trial should be conducted to further evaluate the efficacy of TCM treatment for mastitis.

## 7. Conclusions

The present study provides preliminary clinical evidence supporting the prescription patterns of CHM products in women with mastitis. Approximately 5.1% of women with mastitis received CHM as complementary treatment. Xian-Fang-Huo-Ming-Yin is the most frequently prescribed Chinese herbal formula in these cases. Further well-designed, clinical trials could be developed to evaluate the effectiveness of TCM for mastitis.

## Figures and Tables

**Table 1:** Demographic characteristics of the patients with mastitis by Chinese herbal medicine use.

	CHM user (*n* = 437)		Non-CHM user (*n* = 8,094)		*p*
Age at diagnosed mastitis (years), mean (SD)	33.3	(5.7)	32.9	(6.8)	0.173
20–29	110	(25.2)	2,648	(32.7)	<0.001
30–39	267	(61.1)	4,011	(49.6)	
40–49	60	(13.7)	1,435	(17.7)	
Monthly insurance premium (TWD)					0.014
≥45,801	8	(1.8)	139	(1.7)	
28,801–45,800	17	(3.9)	349	(4.3)	
15,841–28,800	59	(13.5)	1,460	(18.0)	
<15,840	48	(11.0)	1,155	(14.3)	
Dependent	305	(69.8)	4,991	(61.7)	
Urbanization level					0.257
Urban	276	(63.2)	4,896	(60.5)	
Suburban	63	(14.4)	1,097	(13.5)	
Rural	98	(22.4)	2,101	(26.0)	

CHM, Chinese herbal medicine; TWD, Taiwan dollars.

**Table 2:** Ten most prescribed single Chinese herbs for mastitis (total prescription numbers = 7,693).

CHM			Frequency	%	Average duration for prescription (days/visit)	Average daily dose (g)	CHM effects	Possible pharmacological effects	Reference
Chinese name	Scientific name	English name							
Mai Men Dong	*Ophiopogon japonicus* (Thunb.) Ker Gawl.	Dwarf lilyturf root	63	22.3	7.0	1.2	Nourishes vital essence, removes heat from the heart, and removes dryness from the lung	Anti-inflammation	[10]
Pu Gong Yin	Taraxacum mongolicum Hand.-Mazz.	Mongolian dandelion herb	22	7.8	6.	1.8	Clears heat, resolves toxins, and expels node	Anti-inflammation, analgesic effects	[11]
Wang Bu Liu Xing	Vaccaria hispanica (Mill.) Rauschert	Cowherb seed	10	3.5	6.3	1.0	Activates blood, promotes milk secretion, disperses swelling, and reduces sores	Anti-inflammation, analgesic effects	[12]
Xiang Fu	*Cyperus rotundus L.*	*Cyperus* rhizome	9	3.2	6.7	1.1	Soothes the liver and rectifies *Qi*	Anti-inflammation	[13]
Chuan Lian Zi	*Melia azedarach* L.	Sichuan chinaberry fruit	8	2.8	6.0	1.3	Rectifies *Qi*, relieves pain, and clears liver-heat	Anti-inflammation, analgesic effects	[14]
Lu Lu Tong	*Liquidambar formosana* Hance	Beautiful sweetgum fruit	8	2.8	6.1	1.3	Dispels wind and frees the channels	Anti-inflammation	[15]
Yu Jin	*Curcuma phaeocaulis* Valeton	Curcuma root	7	2.5	6.9	1.0	Promotes blood circulation for relieving pain, invigorates the flow of *Qi* for soothing depressed liver, and clears away heat in the blood and heart	Anti-inflammation, analgesic effects	[16]
Tian Hua Fen	*Trichosanthes kirilowii Maxim.*	*Trichosanthes* root	6	2.1	5.7	1.1	Clears heat and engenders fluid, disperses swelling, and expels pus	Anti-inflammation	[17]
Zhe Bei Mu	*Fritillaria thunbergii* Miq.	Thunberg fritillary bulb	6	2.1	6.2	1.4	Resolves heat-phlegm	Inhibits IL-6, IL-8, TNF-alpha, and MAPK pathways	[18]
Jin Yin Hua	*Lonicera japonica Thunb.*	Honeysuckle flower bud	6	2.1	6.3	2.2	Dispels wind-heat and removes heat and toxic substances	Antiviral	[19]

CHM, Chinese herbal medicine.

**Table 3:** Ten most prescribed Chinese herbal formulae for mastitis (total prescription numbers = 7,693).

Chinese herbal formulae name	Ingredients	Frequency	%	Average duration for prescription (days/visit)	Average daily dose (g)	Effects
Xian Fang Huo Ming Yin	*Lonicera japonica* Thunb., *Fritillaria thunbergii* Miq., *Gleditsia sinensis* Lam., *Angelica sinensis* (Oliv.) Diels, *Trichosanthes kirilowii* Maxim., *Boswellia sacra* Flueck., *Saposhnikovia divaricata* (Turcz.) Schischk., *Paeonia lactiflora* Pall., *Commiphora myrrha* (Nees) Engl., *Manis pentadactyla* Linnaeus, *Citrus reticulata* Blanco, *Glycyrrhiza uralensis* Fisch.	28	18.2	6.4	4.5	Clears heat, resolves toxins, disperses swelling, promotes suppuration, invigorates blood, and relieves pain
Jia Wei Xiao Yao San	*Paeonia suffruticosa* Andrews, *Gardenia jasminoides* J.Ellis, *Paeonia lactiflora* Pall., *Bupleurum chinense* DC., *Mentha haplocalyx* Briq., *Angelica sinensis* (Oliv.) Diels, *Atractylodes macrocephala* Koidz., *Poria cocos* (Schwein.) F.A.Wolf, *Glycyrrhiza uralensis* Fisch., *Zingiber officinale* Roscoe	14	9.1	6.6	6.9	Clears heat to cool the blood, soothes the liver, and releases depression
Chai Hu Shu Gan San	*Bupleurum chinense* DC., *Ligusticum striatum* DC., *Cyperus rotundus* L., *Paeonia lactiflora* Pall., *Citrus reticulata* Blanco, *Glycyrrhiza uralensis* Fisch., *Citrus* × *aurantium* L.	13	8.4	6.3	3.4	Soothes the liver and regulates *Qi*
Xiao Yao San	*Paeonia lactiflora* Pall., *Bupleurum chinense* DC., *Mentha haplocalyx* Briq., *Angelica sinensis* (Oliv.) Diels, *Atractylodes macrocephala* Koidz., *Poria cocos* (Schwein.) F.A.Wolf, *Glycyrrhiza uralensis* Fisch., *Zingiber officinale* Roscoe	8	5.2	8.2	5.2	Soothes the liver and resolves constraint, nourishes blood, and fortifies the spleen
Shi Liu Wei Liu Qi Yin	*Angelica sinensis* (Oliv.) Diels, *Aucklandia lappa* DC., *Ligusticum striatum* DC., *Magnolia officinalis* Rehder & E.H.Wilson, *Platycodon grandiflorus* (Jacq.) A.DC., *Areca catechu* L., *Astragalus membranaceus* (Fisch.) Bunge, *Citrus* × *aurantium* L., *Panax ginseng* C.A.Mey., *Lindera aggregata* (Sims) Kosterm., *Cinnamomum cassia* (L.) J.Presl, *Perilla frutescens* (L.) Britton, *Angelica dahurica* (Hoffm.) Benth. & Hook.f. ex Franch. & Sav., *Saposhnikovia divaricata* (Turcz.) Schischk., *Glycyrrhiza uralensis* Fisch., *Paeonia lactiflora* Pall.	4	2.6	6.2	6.5	Promotes the movement of *Qi* and blood, disperses stagnation, expels pus, and dissipates swelling
Wu Wei Xiao Du Yin	*Lonicera japonica* Thunb., *Taraxacum mongolicum* Hand.-Mazz., *Chrysanthemum indicum* L., *Viola philippica* Cav., *Malva verticillata* L.	4	2.6	8.2	4.9	Clears heat, relieves toxicity, cools the blood, reduces swelling, and reduces external carbuncles and furuncles
Su Wu Tang	*Ligusticum striatum* DC., *Angelica sinensis* (Oliv.) Diels, *Paeonia lactiflora* Pall., *Rehmannia glutinosa* (Gaertn.) DC.	4	2.6	6.5	4.6	Tonifies and harmonizes blood
Shao Yao Gan Cao Tang	*Paeonia lactiflora* Pall., *Glycyrrhiza uralensis* Fisch.	4	2.6	6.2	4.5	Relaxes tension to relieve pain
Xiao Chai Hu Tang	*Bupleurum chinense* DC., *Scutellaria baicalensis* Georgi, *Panax ginseng* C.A.Mey., *Pinellia ternata* (Thunb.) Makino, *Glycyrrhiza uralensis* Fisch., *Zingiber officinale* Roscoe, *Ziziphus jujuba* Mill.	3	2.0	5.0	6.0	Harmonizes and releases the lesser yang (shaoyang)
Si Ni San	*Bupleurum chinense* DC., *Citrus* × *aurantium* L., *Paeonia lactiflora* Pall., *Glycyrrhiza uralensis* Fisch.	3	2.0	5.7	4.2	Soothes the liver and regulates the spleen

## Data Availability

The data used to support the findings of this study are included within the article.

## Abbreviations

CHM: Chinese herbal medicine
ICD-9-CM: International Classification of Diseases, 9th Revision, Clinical Modification
LHID: Longitudinal Health Insurance Database
NHIRD: National Health Insurance Research Database
TCM: Traditional Chinese medicine
TWD: Taiwan dollars.
